# Expression of toll-like receptor 4 in uvea-resident tissue macrophages during endotoxin-induced uveitis

**Published:** 2009-03-30

**Authors:** Wei Chen, Xiaofeng Hu, Li Zhao, Shang Li, Hong Lu

**Affiliations:** Department of Ophthalmology, Beijing Chaoyang Hospital, Capital Medical University, Beijing, China

## Abstract

**Purpose:**

To investigate the dynamics and distribution of toll-like receptor 4 (TLR4)-positive cells and resident tissue macrophages in the uvea during endotoxin-induced uveitis (EIU) in Wistar rats.

**Methods:**

Wistar rats (n=40) received a footpad injection of 200 μg of *Vibrio cholera* lipopolysaccharide (LPS), and the intensity of anterior segment inflammation was evaluated after the LPS injection. Ten rats each were killed 6, 12, 24 and 48 h after injection. Ten normal Wistar rats were killed as controls (0 h). The iris-ciliary body complex and choroids from each eye were removed and subdivided into segments. Immunohistochemical localization of TLR4 and a resident tissue macrophage marker, cluster of differentiation 163 (CD163), was performed on whole mount isolated iris-ciliary body complexes and choroids. TLR4^+^ and CD163^+^ cells in the iris were manually counted, and the cell density (cells/mm^2^) was calculated. The distribution patterns and phenotypes of cells expressing these two proteins were further characterized by double-labeled immunofluorescence studies.

**Results:**

The iris-ciliary body complex did not express TLR4 in normal rats. TLR4^+^ cells were detectable in the iris stroma 6 h after injection, and the number significantly increased (p<0.001 by one-way ANOVA) 12, 24, and 48 h after injection. The morphology of TLR4^+^ cells hardly changed 12–48 h after injection. CD163 was expressed in the uvea in all rats. During the inflammatory response phase (0–48 h after injection), the proportion of CD163^+^ tissue macrophages having a round morphology increased (p<0.001 by one-way ANOVA) concurrently with a decrease in the proportion of dendritiform CD163^+^ cells. These changes occurred mainly in the macrophages located in the stroma bordering the iris endothelial layer. Double-labeling immunofluorescence demonstrated the co-expression of TLR4 and CD163 in round stroma cells with TLR4 located at the cell membrane and CD163 in the cytoplasm. TLR4^+^ cells could not be detected in choroids in any of the rats.

**Conclusions:**

The results of the present study indicate that TLR4 expression increased in the iris and iris tissue macrophages expressed TLR4 during EIU. This has significant implications for the understanding of ocular inflammation and for interpreting the potential role of Gram-negative bacteria in the pathogenesis of acute anterior uveitis.

## Introduction

Uveitis is an intraocular inflammatory disease which is potentially sight threatening. Uveitis can be classified as anterior uveitis, intermediate uveitis, posterior uveitis, or panuveitis [1]. Anterior uveitis is the most common form of uveitis. The etiology of uveitis is unclear, but it is speculated to be an autoimmune response resulting from a breakdown in the normal state of ocular immune privilege [2]. Extensive clinical and experimental evidence support the role of particular Gram-negative bacteria or their lipopolysaccharides (LPS) in the pathogenesis of noninfectious, immune-mediated acute anterior uveitis (AAU), particularly those that are associated with human leukocyte antigen-B27 (HLA-B27; see a recent review by Chang et al. [3]). Furthermore, in endotoxin-induced uveitis (EIU), a well established animal model of AAU [4], an injection of LPS to certain susceptible strains of rodents induces an acute and preferential inflammation of the iris and ciliary body that closely resembles AAU in humans.

Toll-like receptors (TLRs) are a family of type I transmembrane pattern-recognition receptors (PRRs) that are essential in the recognition of the highly conserved pathogen-associated molecular patterns (PAMPs) [5,6]. Stimulation of the TLR by its specific PAMP results in the activation of an immunostimulatory and immunomodulatory cell signaling pathway that is essential for innate immunity and the activation of the adaptive immune response [7,8]. It is now well known that TLR4 is the primary signaling receptor for LPS-specific recognition and cellular activation [9]. TLR4 expression has been demonstrated on macrophages, peripheral blood monocytes, dendritic cells (DCs), and dermal vascular endothelial cells as well as in various tissues [10-13]. One of the earliest phagocytes to respond to infection is the tissue macrophage, which originates as monocytes in the peripheral blood system [14]. Activation of TLR4^+^ macrophages by LPS induces various proinflammatory cytokines, chemokines, and antimicrobial activities. Therefore, these innate immune cells are expected to be able to respond rapidly to LPS of Gram-negative bacteria. Macrophages play a key role in the pathogenesis of EIU [15].

Cluster of differentiation 163 (CD163) is a group B cysteine-rich scavenger receptor that is exclusively expressed by cells of the monocyte–macrophage lineage [16] and functions as a scavenger receptor for hemoglobin–haptoglobin complexes [17]. The activation of human CD163 results in the production of pro- and anti-inflammatory cytokines, suggesting a potential role of CD163 in host defense and/or inflammation [18,19]. Rat CD163 is a marker for most mature tissue macrophages and can trigger the production of pro-inflammatory mediators in macrophages [20]. McMenamin and Crewe [21] have previously reported the kinetics of tissue macrophages in the iris during EIU and demonstrated that the number of cells with round-pleiomorphic morphology increased 0–24 h after LPS injection.

Chang and colleagues [2,22] have reported the expression of TLR4 and its associated lipopolysaccharide receptor complex in resident antigen-presenting cells in the human uvea in vivo. Human ciliary body non-pigmented epithelial cells also showed TLR4 expression [23]. Recently, de Kozak et al. [24] detected the expression of TLR4 in the epithelium of the Lewis rat iris and in several ectodermal dysplasia 1 (ED1)-positive cells adjacent to this epithelial layer during EIU.

The uveal tract has a particular predilection for involvement in intraocular inflammation, and Gram-negative bacteria may also play a role in the pathogenesis of AAU. The purpose of the present study was to investigate the dynamics and distribution of TLR4^+^ cells and resident tissue macrophages in the iris during the progression of EIU in Wistar rats.

## Methods

### Animals

Adult male pathogen-free Wistar rats (8–10 weeks old, weighing 150–200 g) were obtained from the Capital Medical University Animal Resource Centre (Beijing, China) and housed in 12 h light/12 h dark cycles. Throughout this study, all procedures adhered to the Institute for Laboratory Animal Research guidelines (Guide for the Care and Use of Laboratory Animals). Fifty animals were used in the study. Animals were randomly divided into five groups (n=10 per group) for the following time points: before LPS injection (0 h; control group) and 6, 12, 24, and 48 h after LPS injection.

### Immunization and tissue preparation

Endotoxin-induced uveitis was induced as previously described [21]. Lipopolysaccharide (*V. cholera*,  classical Biotype, serotype Ogawa) was kindly provided by Lanzhou Institute of Biologic Products (Lanzhou, China). Each animal (except the control group) received a single injection in one rear footpad of 200 μg LPS dissolved in 250 μl sterile saline (NS). The eyes were examined by slit microscope before the injection and at 2 h intervals after the injection, and the intensity of anterior segment inflammation was evaluated. As previously described [19], the severity of uveitis was graded from 0 to 4 by a masked investigator as follows: 0=no inflammation; 1=discrete vasodilatation of the iris and the conjunctiva vessels; 2=moderate dilatation of the iris and the conjunctival vessels with moderate flare in the anterior chamber; 3=intense iridal hyperemia with intense flare in the anterior chamber; and 4=the same clinical signs as 3 with fibrinous exudates in the pupillary area. Animals were killed either before LPS injection (0 h; control group) or 6, 12, 24, or 48 h after the injection. Rats were perfused, and whole mounts of the choroids and iris-ciliary body complexes were prepared as described below.

### Single-labeling immunohistochemistry of uvea whole mounts

Immunohistochemistry of uvea whole mounts was performed according to previously reported methods [25,26]. After the eyes were observed using a slit microscope, the rats were killed by an overdose of pentobarbital (100 mg/kg bodyweight; Sigma, St. Louis, MO) and flushed through the left ventricle of the heart with 250–350 ml of cold (4 °C) phosphate-buffered saline (PBS), 1 IU heparin per ml of PBS, and 250 ml of cold (4 °C) 4% paraformaldehyde. Eyes were enucleated and fixed in 4% paraformaldehyde for 1–2 h. The iris–ciliary body complex and choroid were gently dissected from the eyes and stored in PBS at –80 °C. The tissues were defrosted for 3 min at 37 °C before use.

Polyclonal antibody TLR4 was purchased from Santa Cruz (sc-30002, rabbit polyclonal antibody; Santa Cruz Biotechnology, Santa Cruz,CA). Monoclonal antibody CD163 (MCA342GA) and mouse IgG1 (MCA1209) were purchased from Serotec (Oxford, UK), and affinity purified normal rabbit IgG was purchased from Boster (Boster Biotechnology, Wuhan, China). Other reagents used were PV-6000 (polymer detection system for immuno-histological staining; Zhongshan Goldbridge Biotechnology, Beijing, China) and a 3, 3′-diaminobenzidine tetrahydrochloride substrate kit (DAB; Zhongshan Goldbridge Biotechnology, Beijing, China).

Endogenous peroxidase activity in the whole mounts was blocked by 0.3% H_2_O_2_ for 30 min at room temperature. After three washes with PBS (10 min each), tissue pieces were incubated in small, sealed glass vials containing pre-warmed 20 mM EDTA tetrasodium for 30 min at 37 °C. Whole mounts were then placed in individual wells in a flat-bottomed 24 well plate and quickly rinsed with PBS three times at 10 min per rinse. Increased permeability of the tissue was achieved by replacing PBS with a 0.1% solution of Tween-20 in PBS plus 0.5% Triton X-100. The whole mounts were incubated with 3% bovine serum albumin (BSA)/PBS for 30 min at room temperature to block non-specific binding. Tissues were then incubated with the primary antibodies appropriately diluted in 3% BSA/PBS overnight at 4 °C. The following primary monoclonal antibodies against the indicated specificity in rat tissues were used: anti-TLR4 (2 μg/ml) and anti-CD163 (2 μg/ml). After three washes with PBS, the tissues were incubated with PV-6000 secondary antibodies for 30 min at room temperature. After three further washes in PBS, the tissues were incubated with DAB. The reaction was stopped once suitable color had developed or after a maximum of 10 min. The whole mounts were placed in clean glass vials and dehydrated through an ascending alcohol and xylene gradient and then placed in a drop of mounting medium (Balsam neutral) and xylene on a microscope slide. As a positive control, rat spleen cryostat sections were stained for TLR4. Negative controls were performed by replacing the primary antibody with species-matched and isotype-matched antibodies at the same concentrations of the primary antibody.

### Double-labeled immunofluorescence

Double-labeled immunofluorescence was performed by serially incubating the tissues with two primary antibodies of different species. After blocking non-specific binding with 10% normal goat serum, the tissues were incubated with the first primary antibody, rabbit anti-rat TLR4 polyclonal antibody (4 μg/ml) overnight at 4 °C. After they were washed with PBS, tissues were incubated with fluorescein-conjugated AffiniPure goat anti-rabbit IgG (Zhongshan Goldbridge Biotechnology) for 2 h in the dark at room temperature. All subsequent steps were performed with the tissues protected from light. The tissues were washed with PBS and then incubated with the second primary antibody, anti-CD163 (2 μg/ml) overnight at 4 °C. After they were washed with PBS, tissues were incubated with rhodamine-conjugated AffiniPure goat anti-mouse IgG (Zhongshan Goldbridge Biotechnology) for 2 h at room temperature. After three washes with PBS for 10 min each, slides were mounted in 80% glycerin. Negative controls included replacing the first or second primary antibody or both antibodies with species- and isotype-matched irrelevant antibodies.

Slides were examined under a fluorescence microscope (Leica-DM-4000B; Leica, Wetzlar, Germany). Images were captured, and the pairs of images were superimposed for colocalization analysis using image-management software (Adobe Photoshop CS3. 10.0; Adobe Systems, Mountain View, CA). Double immunofluorescence of whole mounts was also examined by inverted confocal laser scanning microscope (Leica-DM-IRE2; Leica).

### Quantitative analysis

The distribution of immunopositive cells within the rat iris whole mounts was used for quantitative analysis. Positively stained cells were classified during counting as either dendritiform (possessing one, two, or more long, thin, cytoplasmic dendritic processes) or non-dendritic, which was either pleiomorphic (irregular with short, broad cytoplasmic extensions) or round-ovoid [27]. Different layers of stained cells could be distinguished in separate focal planes of the whole mounts. All the cells of different layers in one field were counted as the total number of this field. Immunostained cells were counted in the field located in the middle of the iris whole mount. Four separate fields under a microscope with 40X objective lens were counted by a single masked observer, and the mean number of cells/mm^2^ of each rat was calculated. For CD163^+^ cells, the percent of round-pleiomorphic cells of all positive cells was calculated. All the eyes in each group were examined, and mean numbers were calculated for the group. Positive cell density and the percent of round-pleiomorphic CD163^+^cells were analyzed by one-way ANOVA followed by Least Significant Difference Procedure (LSD) tests for multiple comparisons, using SPSS 11.0 (SPSS Inc., Chicago, IL). A p value less than or equal to 0.05 was considered significant. Data are expressed as means±standard deviation (SD). Single asterisks in figures indicates p≤0.001.

## Results

### Clinical manifestation of endotoxin-induced uveitis

**Figure 1:**
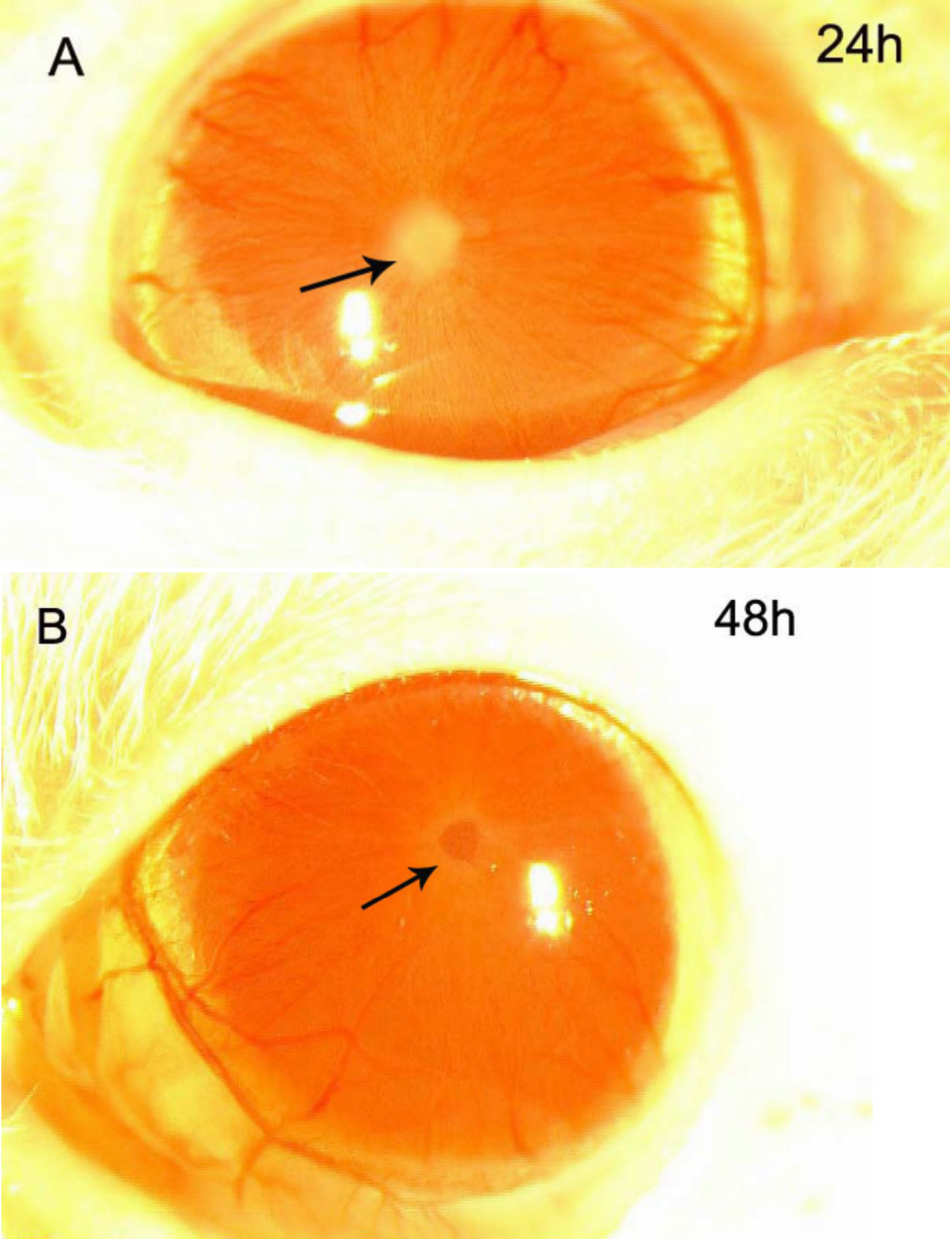
Clinical manifestation of EIU in Wistar rats. **A**: The image shows the eye 24 h after the LPS injection. Note the fibrinous pupillary membrane (arrow). **B**: The image shows the eye 48 h after the LPS injection. The fibrinous pupillary membrane has been absorbed (arrow).

**Figure 2:**
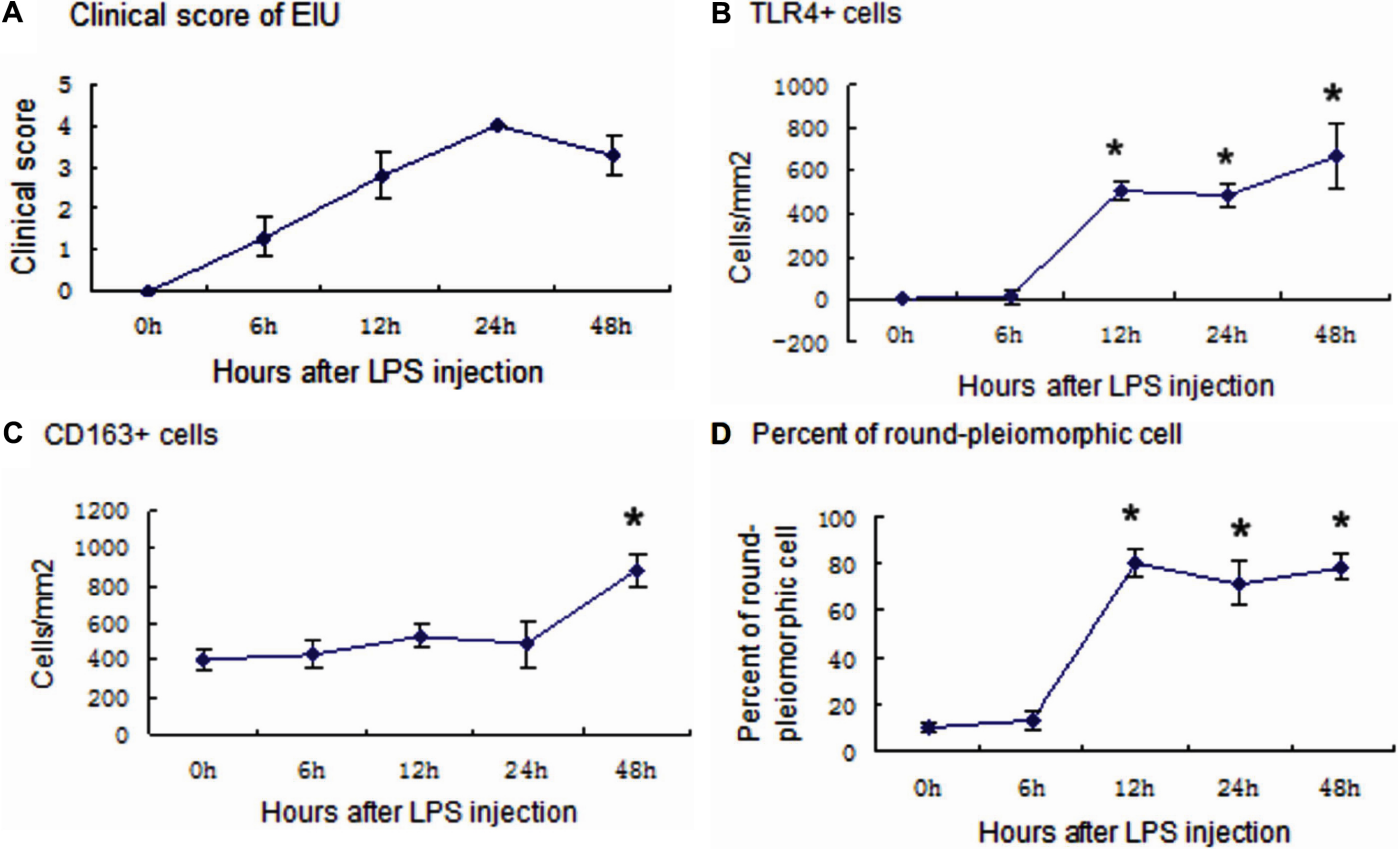
Clinical evaluation and TLR4^+^ and CD163^+^ cells in the iris during the course of EIU. **A**: The clinical score of EIU and inflammation reached their maximum at 24 h. **B**: The density of TLR4^+^ cells in the rat iris had increased greatly 12 h after injection. **C**: There are no obvious changes in the density of CD163^+^ cells in the rat iris within 24 h, but the density had increased 48 h after injection. **D**: Percentage of round-pleiomorphic CD163^+^ cells in the rat iris during the course of EIU is shown in the chart. Significant differences compared with the controls at a level of p<0.001 are shown by asterisks.

Eyes were examined under a slit microscope, and the intensity of the anterior segment inflammation was evaluated. Blood vessels in the iris began to dilate within 4 h after the LPS injection. Examination of the anterior chamber with a slit lamp did not reveal flare or exudation for up to 8 h. At 12–16 h, a fibrinous pupillary membrane could be seen, reaching a maximum at 18–24 h. Hypopyon was also occasionally observed. Generally, inflammation subsided gradually after 24 h, and the exudation had decreased 48 h after LPS injection (Figure 1A,B; Figure 2A).

### Density, distribution, and morphology of TLR4^+^ cells in the iris, ciliary body, and choroid

**Figure 3:**
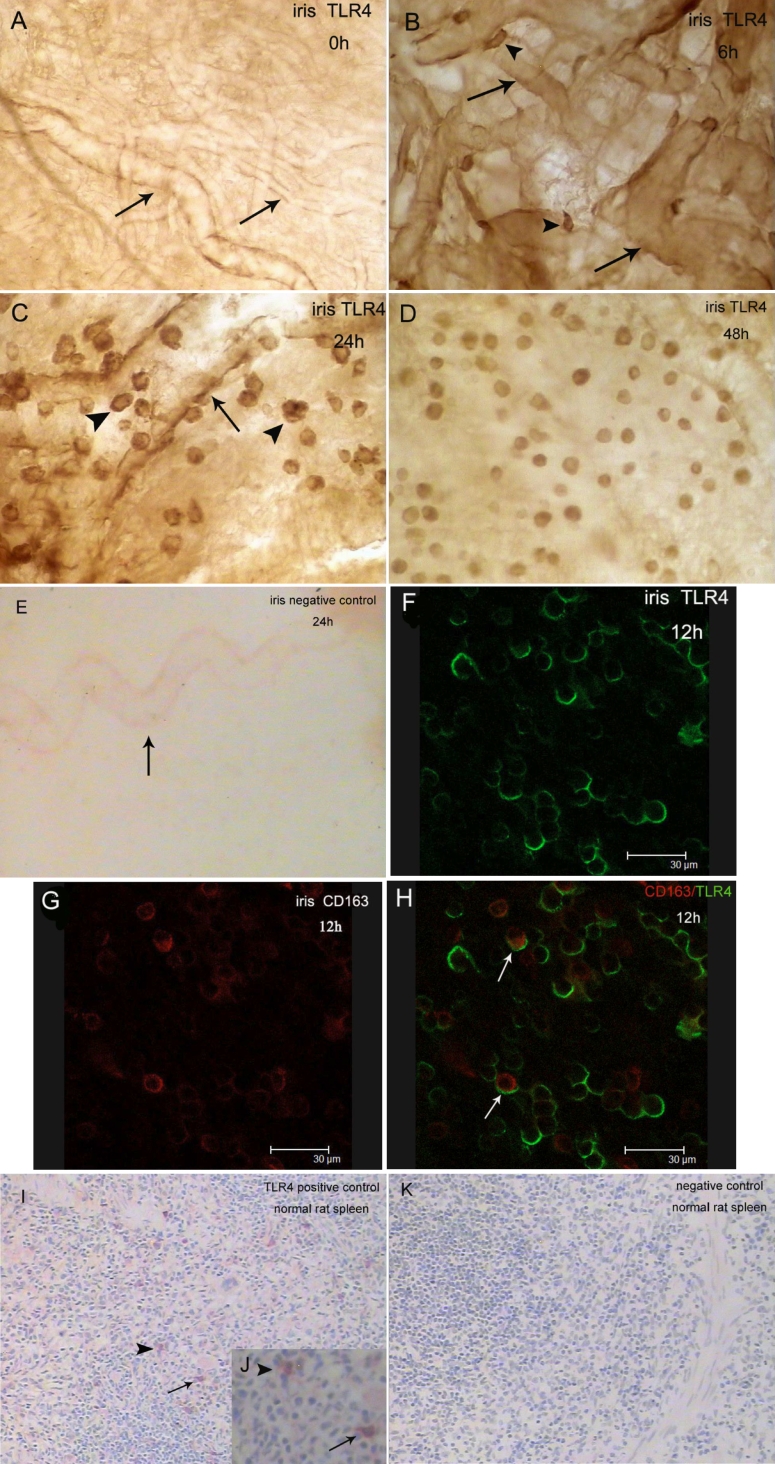
Immunohistochemical studies for TLR4 in iris whole mounts at different times after LPS injection. The dynamics of TLR4^+^ cells in the iris during EIU are shown (**A**–**D**). **A**: Positive cells could not be detected in the control rats. Arrows represent blood vessels. **B**: The TLR4^+^ cells possessed pleiomorphic morphology at 6 h. Note the positive cells (arrowhead) that are located adjacent to blood vessels (arrow). **C**: Most of the TLR4^+^ cells possessed round-ovoid morphology at 24 h (arrowhead). Note the positive cells that are located adjacent to blood vessels (arrow). **D**: The TLR4^+^ cells at 48 h are shown. The morphology and distribution of the positive cells were similar to that at 24 h. **E**: No staining was seen in the iris when under identical experimental conditions when replacing of the primary antibody with normal rabbit IgG at the same concentration (negative control). The arrows indicate blood vessels. **F**–**H**: Double immunofluorescence by confocal microscopy revealed co-expression (arrow) of TLR4 (green) and CD163 (red) by resident stromal cells in the iris. **I**: Positive tissue control shows positive staining for TLR4 by a subpopulation of macrophage-like cells in the normal rat spleen. **J**: A higher power view is shown where the arrow and arrowheads represent the same cells as in panel **I**. **K**: No staining was seen in the spleen when using identical experimental conditions but with the replacement of the primary antibody with normal rabbit IgG at the same concentration (negative control). Original magnification: **B**–**D**,**F**–**H**,**J** 400X; **A**,**E**,**I**,**K** 200X.

Lipopolysaccharides can upregulate the expression of TLR4. TLR4 could not be detected in the iris–ciliary body complex in the control group (0 h). At 6 h, a small number of TLR4^+^ cells were detected in the irises of two rats. The weakly positive cells exhibited pleiomorphic morphology and were located near blood vessels (Figure 3B). The number of TLR4^+^ cells increased significantly (p<0.001) in the iris and ciliary body of all rats at 12 h with a density of 506±39 cells/mm^2^ (Figure 2B), and the immunopositive cells were predominantly round-ovoid cells (Figure 3F) whose morphology was hardly altered between 12 and 48 h after the LPS injection (Figure 3C,D). The TLR4^+^ cells were mainly distributed in the stroma of the iris and in the perivascular location. TLR4 could not be detected in the choroid of any Wistar rats.

### Density, distribution, and morphology of CD163^+^ cells in the iris, ciliary body, and choroid

**Figure 4:**
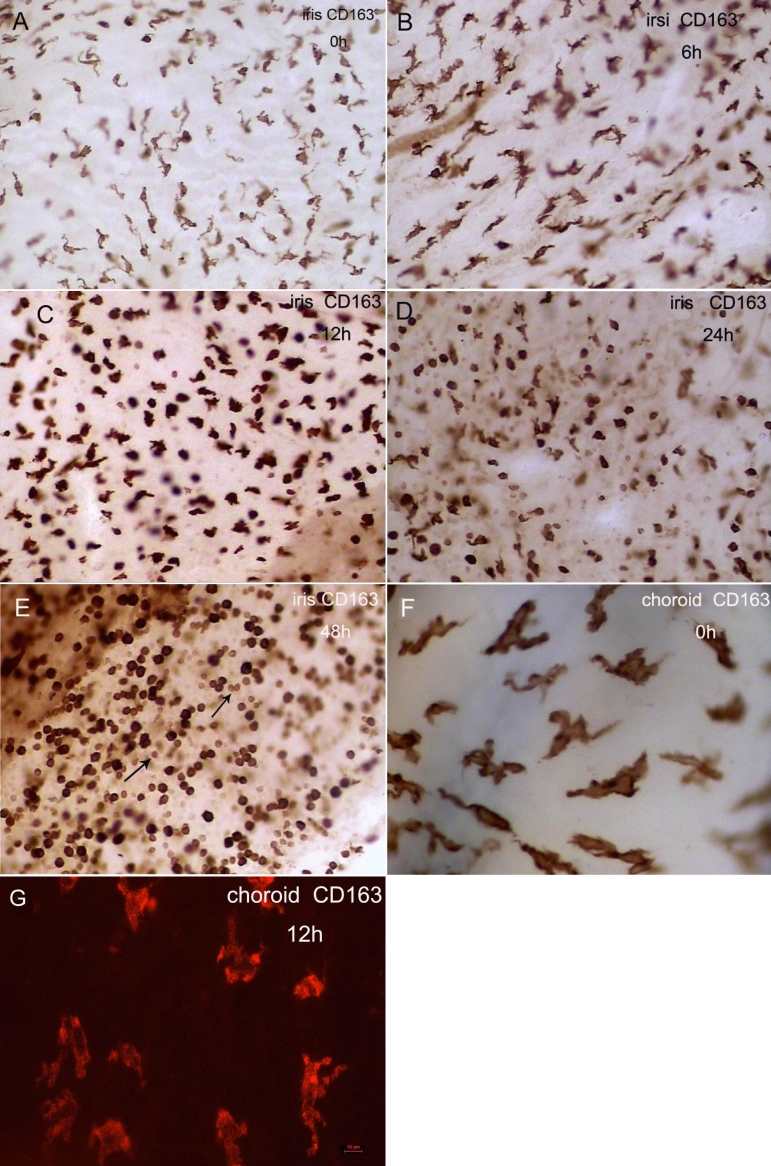
Immunohistochemical studies for CD163 in the iris and choroid whole mounts at different times after LPS injection. **A**–**E**: CD163^+^ resident tissue macrophages in the control rats (**A**) and at 6 h (**B**), 12 h (**C**), 24 h(**D**), and 48h (**E**) after LPS injection in the iris. Note the increase in the number of round-pleiomorphic cells between 12 and 48 h and the numerous small round cells (arrow) at 48 h (**E**). The large dendritiform CD163^+^ cells were unchanged in the peripheral choroid between 0 h (**F**) and 12 h (**G**). Original magnification: **A**–**E** 200X; **F**,**G** 400X.

**Figure 5:**
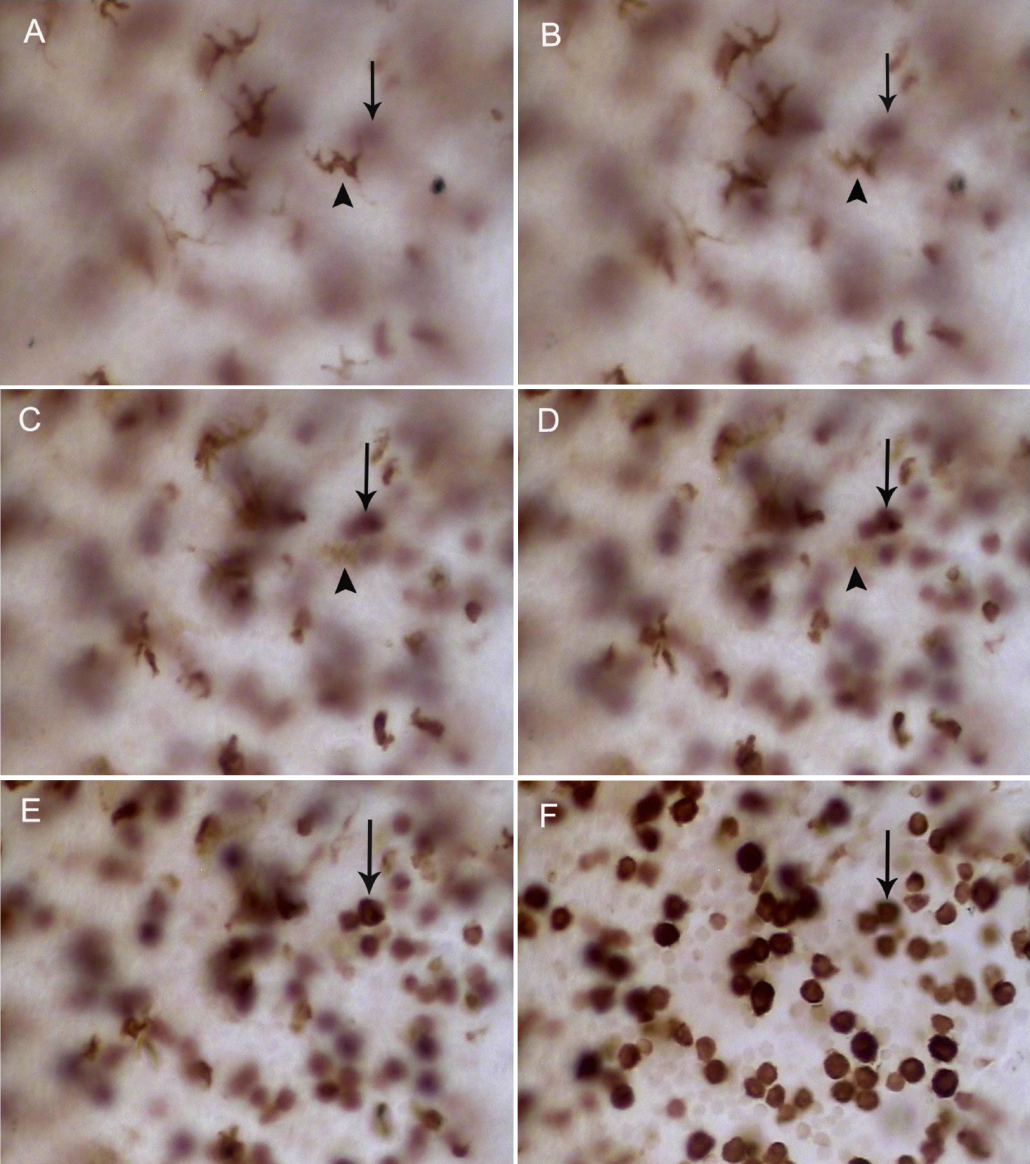
CD163^+^ cells displayed different morphologies at different layers in the iris 48 h after LPS injection. This figure shows different stromal layers from the epithelial to endothelial (from **A** to **F**) in the same field. Dendritiform cells are located in the stroma adjacent to the epithelial layer (**A**) while round-pleiomorphic cells are adjacent to the endothelial layer (**F**). The cells that arrows and arrowheads point to represent the same cells, respectively. Original magnification: **A**–**F** 400X.

The rat CD163 antigen has been identified as an ectodermal dysplasia 2 (ED2) antigen [20], and monoclonal antibody ED2 is a well recognized anti-rat tissue macrophage marker. The network of CD163^+^ tissue macrophages in the current study was predominantly of dendritiform appearance during the first 6 h after LPS injection (Figure 4A,B). At 12 h, the morphology of the positive cells in the iris was markedly changed. The cells possessed fewer dendritic processes, and the proportion of round-ovoid cells was increased (Figure 4C–E). The percentage of cells with round-pleiomorphic appearance increased from 13% in the control rats before injection to approximately 80% of the cell population 12–48 h after injection (p<0.001; Figure 2D). The changes occurred mainly in the macrophages located in the stroma near the iris endothelial layer (Figure 5A–F). During the EIU inflammatory process, there was little change in the total density of CD163^+^ cells (Figure 2C). However, 48 h after injection, small, round, distinctive positive cells were observed (Figure 4E).

In addition, we detected tissue macrophages in the peripheral choroid, which adjoins the ciliary body. The CD163^+^ cells were large and dendritiform in appearance, and the overall density and morphology remained unchanged during EIU (Figure 4F,G).

### Co-expression of TLR4 and CD163 in the iris

To determine the phenotype of TLR4^+^ cells in the iris, a double-immunofluorescence study was performed. Iris whole mounts were double-labeled for TLR4 with a green-emitting fluorochrome (FITC; Figure 3F) and for CD163 with a red-emitting fluorochrome (TRITC, Figure 3G). Some round stromal cells were confirmed to co-express TLR4 and CD163. Confocal microscopy confirmed that TLR4 was located in the cellular membrane while CD163 was located in the cytoplasm (Figure 3H). There were also subpopulations of TLR4^+^/CD163^-^cells and TLR4^-^/CD163^+^ tissue macrophages. No dendritiform CD163^+^ cell coexpressed TLR4.

## Discussion

The results presented here have demonstrated that the expression of TLR4 in the iris is upregulated during endotoxin-induced uveitis. Furthermore, we have shown that some of the TLR4^+^ cells are resident tissue macrophages. These findings have significant implications for our understanding of the innate and adaptive immunity of the eye as well as for the pathogenesis of AAU.

It is becoming increasingly apparent that particular Gram-negative bacteria or their lipopolysaccharides may be important in the pathogenesis of noninfectious immune-mediated HLA-B27-associated AAU [3]. A recent study with patients with AAU also supports this hypothesis [2]. Chang et al. [2] reported that TLR4 stimulation of whole blood in patients with active AAU resulted in a significantly reduced production of pro-inflammatory cytokines such as interleukin 6 (IL-6) and interferon α (IFN-α). This phenomenon was consistent with the observation of a functional state of endotoxin tolerance. Pre-exposure to LPS reduces sensitivity to a subsequent challenge with LPS. This transient functional state of LPS hypo-responsiveness is known as endotoxin tolerance. These findings suggest that patients with active AAU had been recently exposed to LPS.

Endotoxin-induced uveitis is a well established animal model of AAU. Injection of LPS from Gram-negative bacteria at sites remote from the eye induces AAU without significantly affecting other tissues [4,28]. In this study, we detected the upregulated expression of TLR4 in the iris after LPS injection and TLR4 expression preceding the maximal clinical inflammation. The distribution of TLR4, which was predominantly in the iris and the ciliary body, was consistent with anterior segment inflammation in AAU. These results suggest a role for TLR4 in the pathogenesis of AAU. We also show that some TLR4^+^ cells are resident tissue macrophages. Tissue macrophages are one of the earliest phagocytes to respond to infection [14,20]. In the present study, the finding that the TLR4^+^ tissue macrophages are located in the stroma and perivascular locations within the iris suggests that such cells are optimally positioned to assess and to respond to LPS of invasive organisms that have breached the blood–ocular barrier. Activation of TLR4 on macrophages by LPS results in the activation of the transcriptional factor, nuclear factor-κB (NF-κB), via an immunostimulatory intracellular signaling pathway. Consequently, the induction of various proinflammatory cytokines, chemokines, and antimicrobial activities [29] initiates a rapid inflammatory response characterized by the recruitment of leukocytes to the site of infection to eliminate the invading pathogen.

TLR4 expressed on professional antigen presenting cells (APCs) such as macrophages and DCs are a critical link between the innate and adaptive immunity. Chang et al. [22] reported a network of TLR4^+^ APCs, mostly HLA-DR^+^ DCs, in the normal human iris root and the ciliary body. De Kozak and colleagues [24] discovered TLR4 expression in ED1-positive cells (macrophages and dendritic cells) in the iris stroma of EIU. TLR-mediated activation of DCs induces DC maturation with the production of pro-inflammatory cytokines and upregulation of costimulatory and major histocompatibility complex (MHC) molecules to enhance the antigen presenting capacity of DCs [30,31]. Thus, TLR stimulation of APCs leads to the activation and priming of antigen-specific, naive T cells, triggering the adaptive arm of the immune response. Activation of TLR4 in DCs induces production of IL-12, thereby skewing the differentiation toward the T helper cell 1(Th1) type [6,30,31]. Thus, TLRs are important in both triggering and modulating the activation of the adaptive immune response [30,32]. There are various potential mechanisms of Gram-negative bacteria and TLR4 involvement in the pathogenesis of AAU. First, LPS-mediated activation of TLR4 may be one of the earliest initiating factors in the development of AAU [2]. Inappropriate TLR4-mediated activation of the innate and adaptive immune responses within the uvea by LPS may be a major contributing factor in the initiating mechanisms of AAU. TLR4-mediated activation of resident uveal APCs may initiate the breakdown of the ocular immune privilege, resulting in the induction of an autoimmune response [22]. Second, products of tissue inflammation as endogenous ligands for TLR4 may contribute to the perpetuation of uveitis. Intriguingly, such endogenous ligands, including fibronectin, heat shock proteins (HSPs), hyaluronic acid, and host-derived RNA, are released upon tissue damage and cell stress, events that are likely to occur during inflammatory conditions [33]. The activation of cells by endogenous components from distressed or injured cells supports the so-called “Danger Model” proposed by Matzinger [34]. This theory suggests that the immune system is more concerned with damage than with foreignness and is activated by alarm signals from injured tissues rather than by the recognition of non-self. This may be a mechanism for the perpetuation of uveitis and the progression to chronic inflammation because the products of tissue inflammation further stimulate the immune cells that have infiltrated the eye via TLR4. In addition, the heightened ability to respond to LPS in inactive anterior uveitis patients may predispose these individuals to the development of ocular inflammation by LPS-mediated TLR4 activation. Endotoxin tolerance is a transient state [2]. Huhtinen et al. reported that patients with a history of AAU but not with active uveitis have higher functional responsiveness to LPS. The heightened ability to respond to LPS was significant in the presence of a low concentration of LPS (10 ng/ml). At such low concentrations, monocyte activation is mediated specifically via a high-affinity LPS receptor complex, TLR4, myeloid differentiation-2 (MD-2), and cluster of differentiation 14 (CD14). Huhtinen et al. [35] also reported that patients with previous anterior uveitis had smoldering systemic inflammation, which was promoted by innate immunity mechanisms and not by chronic T cell activation.

Here, we report that the normal Wistar rat iris does not express TLR4 whereas Chang et al. [22] had detected TLR4^+^ APCs in the normal human uvea. We found that the TLR4^+^ cells in the Wistar rats were predominantly round-ovoid cells, which was different from the dendritiform positive cells in the normal human iris. This may be due to species differences. The TLR4^+^ cells in two rats 6 h after injection were weakly positive and pleiomorphic, and it is also possible that TLR4 is expressed in the normal rat uvea at low or undetectable constitutive levels. Upregulation of TLR4 from their undetectable constitutive levels in normal rats may occur in response to LPS injection. The pleiomorphic cells in rats 6 h after injection had a range of dendritiform and round appearance, implying that the morphologic changes of TLR4^+^ cells occur during EIU. More study on rats between 6 and 12 h after LPS injection are required to confirm this finding. De Kozak [24] previously reported the expression of TLR4 in the iris epithelium of Lewis rats using cryostat sections. We did not detect TLR4 in the iris epithelium of Wistar rats using iris whole mounts. The different findings may be due to differences between Lewis and Wistar rats or because of the different staining methods used. We used iris whole mount immunohistochemical methods in the present study. The advantages of this approach are the more rapid tissue preparation time, visualization of the morphology of entire cells and cell networks, and the increased sampling of tissue. However, the observation of the iris epithelium cytomembrane in whole mounts is less clear than in sections.

Macrophages have various functions and play a critical role in host defense. However, macrophages are heterogeneous and exhibit a wide range of phenotypes with regard to their morphology, cell surface antigen expression, and function [36]. Rat CD163 (rat ED2 antigen) is a marker for most mature tissue macrophages but is not expressed in rat monocytes [20]. McMenamin [21] previously reported the kinetics of ED2^+^ tissue macrophages in the iris during EIU and demonstrated that the number of cells with round-pleiomorphic appearance increased over a period of 24 h after injection, which was concurrent with the decrease in the proportion of dendritiform ED2^+^ cells. We extended these findings and confirmed in the current study that the cells with shape alterations were only located in the stroma bordering the iris endothelial layer. The layer of tissue macrophages bordering the iris epithelium was not affected by LPS. Yang et al. [37,38] have previously reported that LPS injection induces early adherence of monocytes to rat retinal blood vessels, which was followed by a massive influx of macrophages into the retina. They also detected that ED2^+^ cells showed a variety of morphologic appearances after LPS injection, including large round cells, pleiomorphic cells, and dendritiform cells. Therefore, these round-pleiomorphic positive cells in the present study were probably recently matured tissue macrophages, which originated from blood monocytes. Whether these different morphologic cells have different roles in the onset and development of EIU is not yet known. A large number of round TLR4^–^ tissue macrophages were observed in this study. Whether the TLR4^–^ tissue macrophages and the TLR4^+^ tissue macrophages have different functions was not investigated in the present study. Further studies to examine the phenotype and function of the macrophages are therefore necessary. The present study showed a higher proportion of the round-pleiomorphic cells after LPS injection than in a previous report [21]. This may be due to the higher dose of LPS injection (200 μg of LPS per rat) than that used in the previous study (100 μg of LPS per rat).

In summary, the results of the present study revealed the expression of TLR4 and CD163 in the uvea during EIU. The preferential expression of TLR4 on tissue macrophages within the iris and ciliary body suggests a novel mechanism for the initiating factors and immunopathogenesis of uveitis, particularly HLA-B27-associated AAU. Further studies, including functional studies, are required to identify the roles of these receptors in the context of uveitis.
